# Oncostatin M: Dual Regulator of the Skeletal and Hematopoietic Systems

**DOI:** 10.1007/s11914-023-00837-z

**Published:** 2024-01-10

**Authors:** Natalie A. Sims, Jean-Pierre Lévesque

**Affiliations:** 1 St. Vincent’s Institute of Medical Research, 9 Princes St, Fitzroy, VIC Australia; 2 Melbourne Medical School, The University of Melbourne, Melbourne, VIC Australia; 3 Translational Research Institute, Mater Research Institute – The University of Queensland, 37 Kent Street, Woolloongabba, QLD Australia

**Keywords:** Oncostatin M, Bone, Osteoblast, Osteoclast, Neurogenic heterotopic ossification, Hematopoiesis, Hematopoietic stem cell mobilization, Diabetes, Mobilopathy, Inflammation

## Abstract

**Purpose of the Review:**

The bone and hematopoietic tissues coemerge during development and are functionally intertwined throughout mammalian life. Oncostatin M (OSM) is an inflammatory cytokine of the interleukin-6 family produced by osteoblasts, bone marrow macrophages, and neutrophils. OSM acts via two heterodimeric receptors comprising GP130 with either an OSM receptor (OSMR) or a leukemia inhibitory factor receptor (LIFR). OSMR is expressed on osteoblasts, mesenchymal, and endothelial cells and mice deficient for the *Osm* or *Osmr* genes have both bone and blood phenotypes illustrating the importance of OSM and OSMR in regulating these two intertwined tissues.

**Recent Findings:**

OSM regulates bone mass through signaling via OSMR, adaptor protein SHC1, and transducer STAT3 to both stimulate osteoclast formation and promote osteoblast commitment; the effect on bone formation is also supported by action through LIFR. OSM produced by macrophages is an important inducer of neurogenic heterotopic ossifications in peri-articular muscles following spinal cord injury. OSM produced by neutrophils in the bone marrow induces hematopoietic stem and progenitor cell proliferation in an indirect manner via OSMR expressed by bone marrow stromal and endothelial cells that form hematopoietic stem cell niches. OSM acts as a brake to therapeutic hematopoietic stem cell mobilization in response to G-CSF and CXCR4 antagonist plerixafor. Excessive OSM production by macrophages in the bone marrow is a key contributor to poor hematopoietic stem cell mobilization (mobilopathy) in people with diabetes. OSM and OSMR may also play important roles in the progression of several cancers.

**Summary:**

It is increasingly clear that OSM plays unique roles in regulating the maintenance and regeneration of bone, hematopoietic stem and progenitor cells, inflammation, and skeletal muscles. Dysregulated OSM production can lead to bone pathologies, defective muscle repair and formation of heterotopic ossifications in injured muscles, suboptimal mobilization of hematopoietic stem cells, exacerbated inflammatory responses, and anti-tumoral immunity. Ongoing research will establish whether neutralizing antibodies or cytokine traps may be useful to correct pathologies associated with excessive OSM production.

## Introduction

Bone and its infiltrating vasculature develop together during embryogenesis. In the late stages of embryonic bone development, the vasculature permeates the mineralized cartilaginous structure by transporting osteoclast precursors [1]. These cells, derived from embryonic erythro-myeloid progenitors, migrate via the circulation into the developing bones, fuse to form osteoclasts, and carve a bone marrow (BM) cavity [2]. This nascent BM cavity is highly vascularized, rich in mesenchymal stromal cells, and is rapidly colonized by circulating definitive hematopoietic stem cells (HSC) mobilized from the fetal liver and spleen [3], thereby seeding a hematopoietic BM that will remain the main hematopoietic tissue throughout life.

Hematopoiesis is dependent on both the bone and BM tissues during fetal development and post-natal life. Fetal defects in osteoclast generation lead to failure of HSC niche formation in the BM and relocation of post-natal hematopoiesis to the spleen [4]. After birth, BM stromal cells (BMSC), skeletal stem cells (SSC), and osteoblast lineage cells are key regulators of hematopoiesis through their production of the CXCL12 chemokine and growth factor KIT ligand. Both are necessary for the maintenance of HSC and of more mature and lineage-committed hematopoietic progenitor cells [5–7]. This is a symbiotic relationship: healthy bones require the hematopoietic system to properly remodel and repair via two myeloid populations generated from bone marrow HSC. Firstly, multinucleated osteoclasts which are derived from colony-stimulating factor-1 receptor (CSF1R)-expressing myeloid progenitors which fuse and can recycle by successive cycles of fission [8] into osteomorphs and re-fusion [9]. Second is a unique population of mononucleated osteal macrophages called osteomacs which provide essential support for osteoblast maturation, survival, and function including bone repair [10–13]. Therefore, as soon as the bones are formed in the developing embryo, skeletal, and hematopoietic tissues are anatomically and functionally intertwined.

Osteoimmunology studies the interactions between bone, hematopoietic, and immune cells. It is well known that inflammation driven by immune cells affects both bone and hematopoietic tissues. For instance, sepsis [14], injection of bacterial lipopolysaccharide (LPS) [15], or injection of granulocyte colony-stimulating factor (G-CSF) [11] which is induced in response to sepsis or LPS, all cause rapid bone loss by suppressing endosteal bone formation and increasing osteoclastogenesis. Sepsis, LPS, and G-CSF also deregulate the function of hematopoietic niches in the BM leading to HSC mobilization into the blood and suppression of medullary erythropoiesis [16] and B lymphopoiesis [17]. Bone loss, HSC mobilization, and lymphopenia in response to sepsis or LPS are G-CSF-dependent [15, 16]; however, the suppression of medullary erythropoiesis is not [16]. G-CSF is a hematopoietic growth factor whose main function is to promote the development and maturation of granulocyte progenitors in the BM in steady-state conditions and in response to infections when hematopoiesis shifts from balanced lymphopoiesis, erythropoiesis, and myelopoiesis to mostly myelopoiesis. However, G-CSF has little role in the maintenance of the bone tissue in steady-state, with no bone phenotype being detected in G-CSF or G-CSFR null mice [18]. Beyond G-CSF, infections also induce the expression of a wide array of inflammatory cytokines by myeloid cells including IL-1α and IL-1β, interferons, tumor necrosis factor (TNF), and IL-6, the roles of which on bone have been recently reviewed [19•]. These inflammatory cytokines also play key roles in adapting hematopoiesis to infection and inflammation with a particular role of IL-1 [20] and interferons [21, 22] in mediating HSC proliferation and favoring myelopoiesis at the expense of lymphopoiesis.

**Fig. 1:**
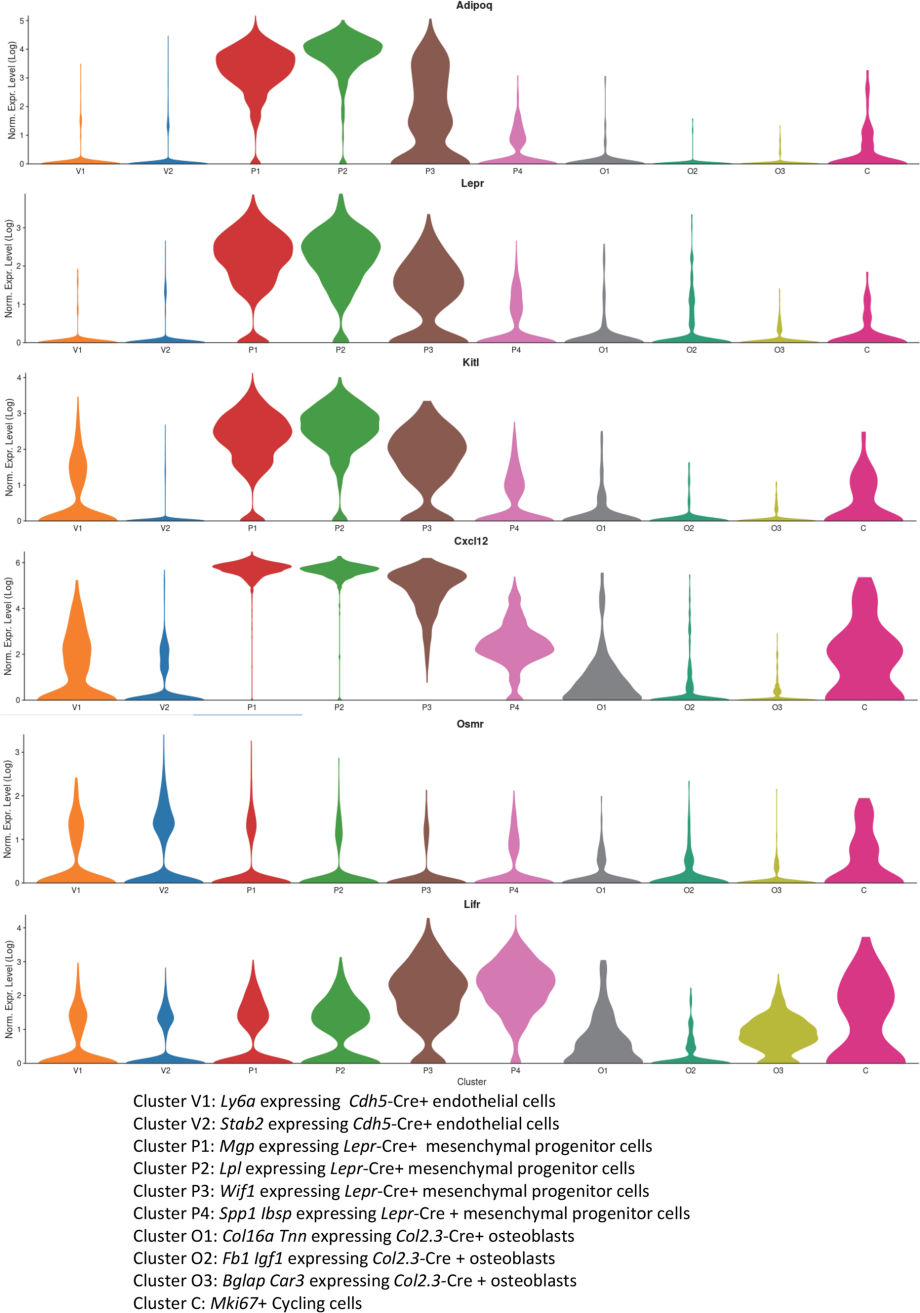
Expression profile of genes encoding OSMR, LIFR, and hematopoietic factors KIT ligand and CXCL12 on non-hematopoietic compartment of the mouse BM. Single-cell RNA sequencing data from mouse BM non-hematopoietic cells are presented as violin plots. Expression levels of *Adipoq*, *Lepr*, *Kitl*, *Cxcl12*, *Osmr*, and *Lifr* mRNA are shown on a Log2 scale in 2 clusters (V1, V2) of endothelial cells identified by labeling with a Cre-inducible fluorescent reporter driven by the VE-cadherin gene (*Cdh5*), 4 clusters of mesenchymal cells identified by labeling with a Cre-inducible fluorescent reporter driven by the leptin receptor gene (*Lepr*), 3 clusters of osteoblasts identified by labeling with a Cre-inducible fluorescent reporter driven by the Col2.3 promoter (*Col2.3*), and a cluster of cycling cells identified by expression of the Ki67 gene (*Mki67*). These plots were generated by using the publicly available nichExplorer date base at https://compbio.nyumc.org/niche/ [28]

Among this plethora of inflammatory cytokines, the IL-6 family cytokine oncostatin M (OSM) is unique as it is expressed at the crossroad of the skeletal and hematopoietic tissues. Indeed, osteocytes, osteoblasts, bone marrow monocytes, macrophages, and granulocytes all express OSM at mRNA and protein levels [23••, 24]. Furthermore, the OSM receptor OSMR is expressed by osteoblasts, osteocytes [24], BM endothelial cells, and mesenchymal stromal cells [23••], which are all essential to bone formation and hematopoiesis. Specifically, the adiponectin (*Adipoq*)-expressing and leptin receptor (*Lepr*)-expressing endosteal SSC (eSSC) responsible for endosteal osteoblast and bone formation reside at the periphery of endothelial sinusoids in the BM [25••]. These *Adipoq* + *Lepr* + eSSC are the same or similar to the *Adipoq* + *Lepr* + BMSC that support HSC maintenance in the BM. Indeed, both these *Adipoq* + *Lepr* + populations express high amounts of CXCL12 and KIT ligand [25••, 26], factors that are essential for HSC maintenance within the BM [5, 6]. These *Adipoq* + *Lepr* + populations also both express the OSM receptor OSMR (Fig. 1). CXCL12, KIT ligand, and OSMR are also expressed by BM endothelial cells (Fig. 1), which are also key components of HSC niches [5, 6]. These observations are consistent with immunohistochemistry of long bone sections from adult mice which reveal that OSMR protein is expressed by endosteal osteoblasts on trabecular and endocortical surfaces [24], discrete BM stromal cells, and BM vascular beds [23••]. The alternate receptor for OSM, leukemia inhibitory factor receptor (LIFR), is also expressed by osteoblasts [27], by more mature BMSC committed to the osteoblast linage expressing osteopontin (*Spp1*) and bone sialoprotein (*Ibsp*) (cluster P4 in Fig. 1), and by endothelial cells (Fig. 1). These findings suggest that OSM is a key regulator of both skeletal and hematopoietic tissues via its OSMR:GP130 and leukemia inhibitory factor receptor (LIFR):GP130 receptor complexes. In this review, we will discuss current understanding of how OSM regulates bone homeostasis and repair as well as hematopoiesis in healthy and diseased conditions.

## OSM and Its Receptor Complexes OSMR:GP130 and LIFR:GP130

OSM is a pleiotropic cytokine first identified by Zarling et al. [29] as a secreted product of the phorbol ester-differentiated U937 histiocytic lymphoma cell line which inhibited proliferation of melanoma-, neuroblastoma-, and lung cancer-derived cell lines. Full-length OSM contains between 239 and 263 amino acids, with an N-terminal signal peptide and a C-terminal pro-domain, which can both be post-translationally cleaved, although the details and biological relevance of this cleavage remain obscure [30]. As all other IL-6 family cytokines, OSM is a long-chain four helix-bundle protein with an up-up-down-down topology. Although OSM amino acid homology is only approximately 50% between mouse and human, and 60% between mouse and rat, the exon–intron structure of the gene encoding OSM is identical in human, mouse, and rat indicating high gene conservation [30]. In all species the *OSM/Osm* gene is located in direct proximity to the gene encoding leukemia inhibitory factor (LIF) suggesting their origins in a gene duplication event [31].

The human OSM gene promoter has been shown to contain response elements to the transcription activator STAT5, which is downstream of several myeloid cytokines such as granulocyte–macrophage colony stimulating factor (GM-CSF) [32]. OSM expression is also induced in vivo in myeloid cells in response to inflammatory stimuli such as G-CSF [23••], prostaglandin E2 [33], or LPS [34, 35••] whereas adiponectin and CCN1 protein have been reported to induce OSM expression in osteoblast cell lines [36, 37]. These further illustrate the functional relationship between inflammation/infection, bone, and hematopoietic homeostasis.

**Fig. 2:**
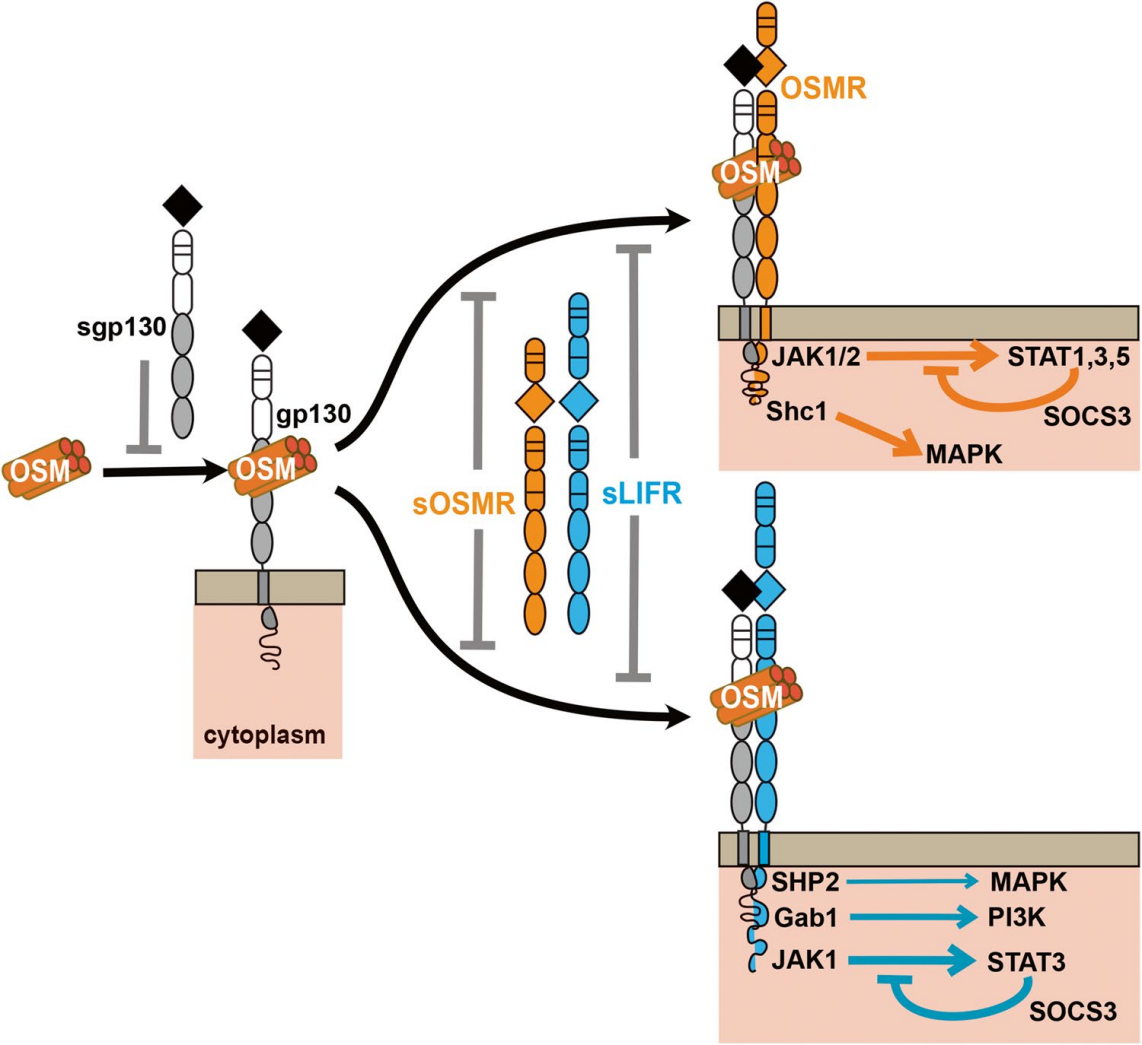
Overview of OSM signaling. OSM binds to transmembrane GP130, an interaction that can be inhibited by soluble GP130 (sGP130). This does not elicit signaling within the cytoplasm. Following this initial interaction, OSM can bind to either OSMR or LIFR, an interaction that is inhibited by soluble forms of both receptors (sOSMR, sLIFR). When OSM interacts with OSMR, the major signaling pathways are initiated by phosphorylation of JAK1 and JAK2 which leads to STAT1, 3, and 5 phosphorylation, a signal under negative feedback from SOCS3. Alternately, SHC1 activates MAPK signaling. In contrast when OSM interacts with LIFR, it can activate MAPK signaling via the SHP2 domain, PI3K signaling via GAB1, and STAT3 signaling via JAK1; the latter is also under negative feedback from SOCS3. The JAK1/STAT3 pathway is the most commonly activated pathway downstream of LIFR binding, followed by PI3K and MAPK

Although all IL-6 family members use the common receptor subunit glycoprotein 130 (GP130), OSM does this in an unusual way by binding to GP130 before recruiting a second receptor subunit to form its signaling complex (Fig. 2) [38]. The other unusual feature of OSM signaling is that, after binding GP130, OSM then heterodimerizes with either the OSM receptor (OSMR) or the leukemia inhibitor factor receptor (LIFR) [39]. In human and rat cells, the affinity of the OSM:GP130 heterodimer for LIFR or OSMR is very similar [39], but in murine cells, OSM binds with much greater affinity for OSMR than LIFR [40]. In mouse and rat cells, human OSM only forms a GP130:LIFR receptor, thereby reflecting LIF biology in these cells when cross-species experiments are conducted [41].

OSMR also associates with the GP130-like receptor IL-31RA (GPL) to form a receptor for the Th2 cytokine IL-31 [42]. IL-31RA null mice exhibit low numbers of immature granulocyte colony forming units, suggesting IL-31 may regulate granulopoiesis and support the osteoclast progenitor pool [43]. However, we found no effect of IL-31 on osteoblast or osteoclast differentiation in vitro [24] and no effects have been reported by others; for this reason, this cytokine will not be discussed further in this review.

There are soluble forms of the OSMR (sOSMR) [44], the LIFR (sLIFR) [45, 46], and GP130 (sGP130) [47]. All arise from alternative splicing, with sGP130 also being produced by proteolytic cleavage [48]. Unlike the soluble form of the IL-6 receptor, which acts as an agonist of its ligand [48], sGP130, sOSMR and sLIFR each act as competitive inhibitors [48] (Fig. 2). All are detected in normal human serum, with sGP130 and sOSMR being in the order of 100 ng/ml and sLIFR being at much lower (4 ng/ml) levels [48]. Both circulating and local production of these inhibitors are likely to modulate OSM signaling, but their abundances within the local bone environment, or in conditions such as heterotopic ossifications and stem cell mobilization, have not been described to date.

OSMR and LIFR primarily activate JAK/STAT signaling (Fig. 2). OSMR binds JAK1 and JAK2 [49], and activates STAT1 [50], STAT3 [51], and STAT5 [50]. OSMR has the unique ability among GP130-binding receptors to also recruit the adaptor protein SHC1 (Src homology and collagen 1) to activate ERK/MAPK signaling [52, 53]. Less studied are reports that OSMR can also activate STAT6 [54], PI3K/Akt [53], and protein kinase C delta (PKCδ) [55]. LIFR activates primarily JAK1/STAT3 signaling [56], but also activates PI3K/Akt [57] and can initiate MAPK signaling directly via its SHP-2 binding domain [30, 57]. Within the cell, STAT3 signaling downstream of both OSMR and LIFR binding and MAPK signaling downstream of LIFR are under negative feedback from Suppressor of Cytokine Signaling 3 (SOCS3) [58].

## OSM Function in Regulating Skeletal Bone Homeostasis

OSM is expressed at all stages of osteoblast differentiation, including by BMSC, matrix-producing osteoblasts, osteocytes, and bone lining cells [24, 59]. These osteoblast lineage cells express all three receptor subunits utilized by OSM (GP130, OSMR, and LIFR) and respond to OSM treatment with increased STAT1, STAT3, and STAT5 phosphorylation [24]. Osteoclast precursors and mature osteoclasts express neither OSMR nor LIFR [24, 27].

Manipulations that increase bone formation stimulate OSM expression and signaling. For example, OSM and OSMR are both significantly upregulated in whole bone samples (including marrow) from rats subjected to mechanical loading [60]. Treatment with parathyroid hormone (PTH) and PTH-related protein (PTHrP) also increases OSMR mRNA in bone samples and in cultured osteoblasts [61•].

Like other IL-6 family members, such as IL-11, IL-6, LIF, and cardiotrophin 1 (CT-1), OSM promotes osteoclast differentiation in vitro [62]. All these cytokines stimulate osteoclast differentiation through indirect means: they stimulate RANKL transcription by supporting cells including BMSC [63] and synovial fibroblasts [64], which then provide RANKL to osteoclast progenitors to induce their differentiation. OSM also induces RANKL expression in osteocytes, but this is not sufficient to fully support osteoclast formation in vitro [65]; again, this is similar to observations made for IL-6 [65].

OSM is a stronger stimulus of osteoclast formation than LIF, a finding first described in the 1990s [62]. This has recently been attributed to the unique ability of OSM to induce expression of the SHC1 adaptor protein [66]. In that study, activation of STAT3 and ERK/MAPK by OSM in calvarial osteoblasts was amplified by the adaptor protein SHC1, and SHC1 inhibition significantly reduced the ability of these cells to support osteoclast formation by production of RANKL [66]. Recent work has shown that, in addition to stimulating RANKL expression in primary calvarial osteoblasts, OSM and IL-6 *trans-*signaling (but not LIF) also induce expression of WNT16 [67], an osteoclast inhibitor with particular importance for protecting the structure of cortical bone [68]. WNT16 was also found to be strongly expressed in cells on periosteal surfaces [67], so this may provide a mechanism to explain why the OSMR null mice exhibit widened metaphyses and a mild Erlenmeyer flask morphology [24], features commonly associated with osteoclast deficiency on the periosteum [69].

In addition to its role as a stimulus of osteoclast formation, OSM promotes osteoblast differentiation in vitro [24, 70, 71] and stimulates bone formation in vivo, either when administered as a recombinant peptide [24] or when increased by transgenic overexpression [72]. There are at least two mechanisms of action. Firstly, OSM promotes stromal cell commitment to osteoblast differentiation rather than adipogenesis [24]. This is likely mediated by induction of the pro-osteoblastic transcription factors C/EBPβ and C/EBPδ [24], and by inhibition of the pro-adipogeneic transcription factor ZFP467 [73]. OSM also suppresses expression of the bone formation inhibitor sclerostin in osteocytes [24, 74].

Consistent with the stimulatory effects of OSM on osteoblast and osteoclast differentiation, mice with global OSMR deletion had low osteoclast numbers, low osteoblast numbers, impaired bone formation on trabecular surfaces, and increased marrow adiposity [24]. Of note, these effects of germinal OSMR gene deletion are not mediated directly by osteoclasts or osteomacs as neither cell type expresses OSMR [23••] (http://biogps.org/#goto=genereport&id=18414), but rather by mesenchymal progenitor cells, osteoblast lineage cells, and endothelial cells which all express high levels of OSMR (Fig. 1).

**Fig. 3:**
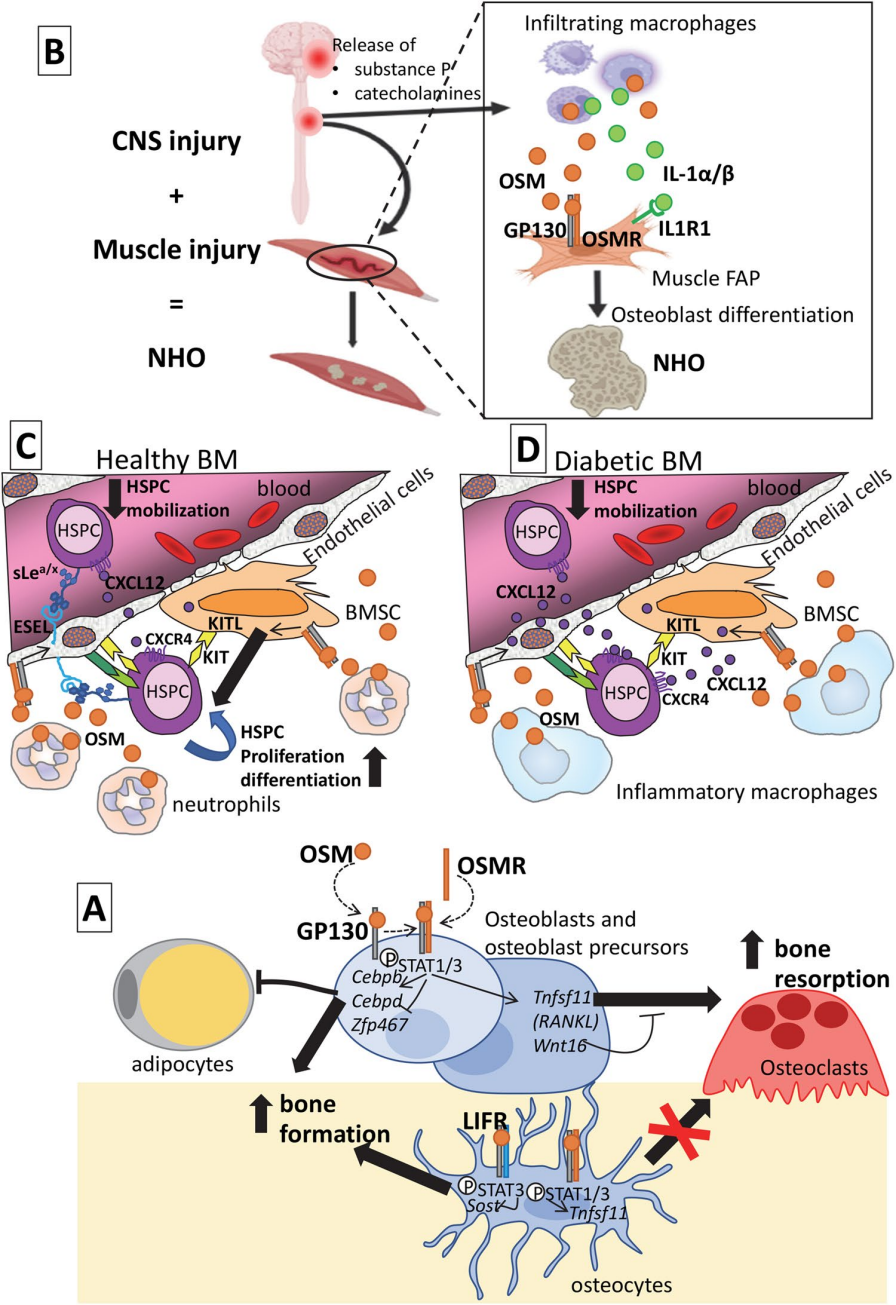
Stage-specific and tissue-specific effects of OSM through multiple receptor complexes. **A** In the bone microenvironment, OSM binds first to GP130 on the cell membrane, then recruits the ligand-specific receptor OSMR. In osteoblast lineage cells, including osteoblast precursors, this activates STAT1 and STAT3 phosphorylation with two distinct outcomes. By inducing expression of the transcription factors *Cebpb* and *Cebpd* and inhibiting *Zfp467*, OSM favors osteoblast commitment and inhibits adipocyte differentiation, thereby promoting bone formation. OSM also acts through the OSMR to induce expression of both *Tnfsf11* (the gene for RANKL) and *Wnt16.* In the presence of osteoclast precursors, this promotes osteoclast differentiation and bone resorption, while WNT16 provides negative feedback. In osteocytes, OSM has different actions depending on receptor usage. Through LIFR (blue), OSM binding primarily leads to STAT3 phosphorylation and inhibition of sclerostin mRNA (*Sost*), a second mechanism that promotes bone formation. Signaling through the OSMR in these cells (orange) induces transcription of *Tnfsf11* (RANKL) but this is insufficient to induce osteoclast generation in vitro. **B** In injured muscles, infiltrating inflammatory macrophages release excessive amount of OSM, IL-1α, and IL-1β in response to neuroendocrine factors released in the circulation following spinal cord injury. OSM binds to OSMR:GP130 and IL-1α/β to IL1R1 expressed on muscle FAP. Through persistent JAK1/2 and STAT3 signaling, FAPs keep proliferating and differentiate into osteoblasts to form NHO. **C** In healthy BM, OSM is released by neutrophils. OSM binds to OSMR:GP130 complex on endothelial cells and BMSC. In endothelial cells, OSMR signals increased expression of E-selectin (ESEL) which increases HSPC retention within the BM, decreases HSPC mobilization, and increases HSPC proliferation via non-canonical E-selectin ligands decorated with sialyl Lewis^a/x^ (sLe^a/x^) sugars. Unknown mediators from BMSC and endothelial cells produced in response to OSM/OSMR signal increases erythroid and megakaryocytic differentiation. **D** The diabetic BM contains inflammatory macrophages releasing excess of OSM. OSM increases release of CXCL12 chemokine by BMSC and endothelial cells which increases HSPC retention within the BM. Mobilization is decreased

The actions of OSM to stimulate bone formation and to stimulate osteoclast formation appear to be mediated, at least in part, through different receptor complexes. In cell culture, osteoblast lineage cells lacking OSMR performed poorly at supporting osteoclast differentiation, even when a stimulus other than OSM was used [24]. Furthermore, in these OSMR deficient cells, OSM either did not [24], or only weakly [75], stimulated RANKL expression by calvarial osteoblasts, indicating this effect is largely OSMR-dependent. Although bone formation was low in OSMR null mice, confirming that OSMR mediates some bone formation response, the OSMR null mice still responded to exogenous OSM treatment with increased bone formation, suggesting an alternative pathway was involved [24]. Support for a second OSMR-independent pathway was also provided by the finding that OSMR null osteocytes also exhibited reduced sclerostin mRNA levels in response to OSM in vitro [24]. This indicated that OSM can promote bone formation through an OSMR-independent pathway. Use of a LIFR-antagonist revealed that OSM suppressed sclerostin through the LIFR [24]. This was surprising, because although human OSM recruits OSMR and LIFR with equal affinity [39], murine OSM binds LIFR with only very low affinity and was presumed to act in mice entirely through OSMR [40]. We concluded that OSM stimulated RANKL expression and osteoclastogenesis through an OSMR:GP130 heterodimer, but stimulated bone formation both by promoting osteoblast commitment at the expense of adipogenesis through *Cebpb*, *Cebpd*, and *Zfp467*, but also by suppressing sclerostin through a LIFR:GP130 heterodimer (Fig. 3).

A follow-up microarray study in murine cells revealed that the OSMR-independent action of murine OSM did not result in activation of a unique set of target genes, but that there was a bias in the signaling initiated: a STAT3-responsive gene signature was stimulated, with little effect on STAT1-responsive genes [75]. Murine OSM induced STAT3 (but not STAT1) phosphorylation in OSMR null cells through LIFR, with minimal phosphorylation of GP130 and LIFR [75]. This suggested that intracellular activation of STAT3 over STAT1 may be anabolic for bone. To test this, STAT1 null mice were crossed with an osteopenic mouse model harboring a GP130-STAT1/3 hyperactivating mutation GP130^Y757F/Y757F^. That strategy of reducing STAT1 availability in the presence of high STAT3 activity prevented development of a low bone mass phenotype [75] and suggested such an approach could be exploited in skeletal pathologies where GP130 signaling is elevated, such as inflammation-induced or metastatic bone loss.

In contrast to OSM, LIF action through LIFR in osteoblasts increases both STAT1 and STAT3 phosphorylation [75], stimulates RANKL [63], and suppresses sclerostin [24]. The mechanism by which murine OSM act through GP130:LIFR to regulate only some gene targets influenced by murine LIF through the same receptor complex may be explained by altered binding conformation, binding affinity, or binding stability, but to date these differences have not been defined.

The initial finding that BM resident macrophages produce OSM has led to several studies indicating that OSM is the mediator through which macrophages support bone formation [76–78]. The earlier mechanistic studies differ in their conclusions as to whether OSM production by macrophages requires M1 type activation [78], contact with BMSCs [77], or M2 type polarization [76]. Nevertheless, they provide a mechanism which may be involved in the activity of resident tissue macrophages to support bone formation both in normal physiology [10] and in fracture healing [79]. It must also be noted that neutrophils are also an important source of OSM [23••]. However, whether OSM produced by neutrophils contributes to bone maintenance is unclear since mice rendered neutropenic through deletion of the G-CSF receptor exhibit no bone phenotype, unless the STAT3 inhibitor SOCS3 is also removed [18]. Additional studies using mice with a floxed *Osm* gene crossed with transgenic mice expressing Cre recombinase controlled by a monocyte/macrophage- or neutrophil-specific promoter will be necessary to clarify a role of neutrophil-produced OSM in regulating skeletal bone homeostasis or repair.

While LIF is a known stimulus of longitudinal bone growth [80], there is little evidence that OSM influences growth plate chondrocytes. However, OSM may contribute to osteoarthritis pathogenesis and OSM treatment is commonly used as a model of cartilage damage in osteoarthritis, since it induces proteoglycan loss and cartilage damage in vitro [81, 82]. Furthermore, local over-expression of OSM in the mouse knee joint induces both cartilage destruction and osteophyte-like periosteal bone formation [83, 84].

## OSM and OSMR in Pathological Heterotopic Ossifications

Traumatic heterotopic ossifications (HO) are pathological extra-skeletal boney growths that develop following a severe trauma. As their name indicates, they are not genetically driven (unlike fibrodysplasia ossificans progressiva) but occur with relatively high frequencies (typically between 5 and 60% depending on etiology) following a variety of trauma such as hip arthroplasty surgery, fractures, severe extended body burns, high-energy multi-trauma or extremity trauma (e.g., following explosive blasts) [85], and severe trauma of the central nervous system in which case they are called neurogenic heterotopic ossifications (NHO) [86]. The pathobiology of these different forms of traumatic HO is not well understood and a variety of models have been designed in rodents to elucidate some of the mechanisms involved in the pathogenesis of traumatic HO [85]. The general consensus is that these traumas exacerbate inflammation in distant injured tissues such as facia, ligaments, subcutaneous, or muscle tissue leading to osteogenic differentiation of resident mesenchymal cells which drive the formation of HO [87, 88•]. How trauma leads to exacerbated inflammation of a distant tissue and how this inflammation leads to osteogenic differentiation remains incompletely understood.

In the particular case of NHO, clinical evidence in victims of traumatic brain injury (TBI) or spinal cord injury (SCI) and experimental evidence in a mouse model of SCI-induced NHO suggest an important role of OSM in the pathogenesis of NHO subsequent to SCI or TBI. OSM concentration was elevated in the blood of TBI and SCI patients developing NHO and OSM protein was also expressed by osteoblasts and osteocytes in NHO biopsies [89••]. In vitro, OSM secreted by LPS-activated human blood monocytes could induce osteoblast differentiation of mesenchymal cells isolated from muscles surrounding NHO [89••]. In a mouse model of SCI-induced NHO, NHO develops in muscles injured by an intramuscular injection of cardiotoxin only when mice have undergone a simultaneous spinal cord transection [90]. In this model, OSM protein was abundantly expressed in injured muscle tissue where NHO developed [89••]. In vitro, mouse OSM also enhanced mineralization of mesenchymal cells isolated from mouse muscles.

The importance of OSM action in NHO pathogenesis following SCI or TBI was shown when NHO development was significantly reduced in *Osmr* null mice compared to wild-type [89••]. At the molecular level, an expression microarray analysis of differentially expressed genes in muscles with or without cardiotoxin-mediated injury and with and without SCI, revealed that SCI triggers in injured muscles the up-regulation of genes related to macrophage activation and inflammation and encoding pro-inflammatory cytokines such as OSM, IL-1α, IL-1β, or TNF [91•]. Using mice lacking the genes encoding the receptors for these cytokines, only OSM and IL-1 were found to contribute to NHO development as mice lacking the *Osmr* gene or *Il1r1* gene had decreased NHO development after SCI [89••, 91•]. Furthermore, LPS was found to exacerbate NHO formation in mice in response to SCI and to increase expression of OSM and IL-1β. This result in mice was concordant with the finding that infection with gram-negative *Pseudomonas aeruginosa* was significantly associated with increased NHO incidence in victims of traumatic brain injury [35••].

OSM and OSMR have been found to promote quiescence of muscle satellite cells (the myogenic stem cells in the muscle) and inhibit their myogenic differentiation in vitro and targeted *Osmr* gene in satellite cells was found to delay muscle regeneration and reduce satellite cell recovery following multiple injury with cardiotoxin [92•]. Genetic lineage tracing experiments indicate that the NHO-forming osteoblasts do not arise by transdifferentiation of satellite cells, but are derived from mesenchymal fibroadipogenic progenitors (FAP) residing in the muscle [88•]. This is consistent with multiple reports that OSM induces osteoblast differentiation in mesenchymal cells isolated from a variety of tissues [24, 78, 89••, 93] and promotes skeletal bone formation [24] and repair [94]. Therefore, excessive OSM in injured muscles subsequent to SCI could both inhibit myogenic differentiation and promote osteogenic differentiation and HO development. These findings are consistent with persistent tyrosine phosphorylation of STAT3 for 2 weeks in injured muscles from mice with SCI (which develop NHO) compared to a return to basal levels 7 days after muscle injury in mice without SCI [95•]. Consistent with this, mouse treatment with the small JAK1/2 tyrosine kinase inhibitor ruxolitinib reduced NHO development by approximately 50% in mice with SCI and muscle injury [95•]. These findings are consistent with the finding that strong STAT3 signaling elicited by OSM and OSMR promotes osteoblast differentiation and is anabolic for bone [75]. Whether OSM is involved in the pathogenesis of other forms of traumatic HO or fibrodysplasia ossificans progressiva has not been reported at the time of this review.

## OSM regulates Steady-State Hematopoiesis via OSMR

The role of OSM in regulating hematopoiesis is underpinned by the effect of germinal inactivation of the *Osm* or *Osmr* genes in mice. Both adult *Osm*^−/−^ mice and *Osmr*^−/−^ mice have a mild but significant anemia and thrombocytopenia with reduced numbers of erythroid progenitors, erythroblasts, megakaryocytes, and megakaryocytic progenitors in the BM in steady-state [96–98]. As a consequence, *Osmr*^−/−^ mice have delayed erythrocyte recovery following a hemolytic challenge with phenylhydrazine [96]. This is consistent with the observations that injection of recombinant OSM in wild-type mice increases thrombopoiesis with higher blood platelet numbers and accelerates platelet and erythrocyte recovery after sublethal irradiation [99]. Conversely, in a double-blinded randomized study in healthy humans, injection of a neutralizing anti-OSM antibody caused a mild thrombocytopenia and anemia at the highest antibody dose [100]. OSM signaling also regulates the proliferation of hematopoietic stem and progenitor cells (HSPC) in the BM. Indeed, all populations of HSPC including long-term reconstituting HSC, short-term repopulating HSC, and multipotent progenitors-2 and -3 populations are more quiescent in the BM of *Osmr*^−/−^ mice [23••]. This could explain their mild anemia and thrombocytopenia since the daily erythrocyte and platelet turn-over is high due to their very large numbers in the blood.

Osteoblasts and macrophages are a recognized source of OSM but it is most abundantly expressed by neutrophils in the healthy mouse BM [23••]. Unlike other pro-inflammatory cytokines, a remarkable feature of OSM is that its receptor OSMR is not expressed by leukocytes or HSPC. Indeed, *Osmr* mRNA is undetectable in BM HSPC or blood and BM leukocytes either by qRT-PCR [23••] or by RNA sequencing of sorted hematopoietic cells and leukocytes (https://www.haemosphere.org). Therefore, the OSMR-dependent OSM effects on HSPC and leukocytes must be mediated by the surrounding non-hematopoietic stroma in the BM. Indeed, both qRT-PCR [23••] and single-cell RNA sequencing (https://combio.nyumc.org/niche/) indicate abundant *Osmr* mRNA in BM CD45^−^ lineage^−^ CD31^−^ CD51^+^ PDGFRα^+^ mesenchymal stromal cells and CD45^−^ lineage^−^ CD31^+^ endothelial cells. This is consistent with previous experiments showing that transplantation chimera of wild-type BM cells into lethally irradiated *Osmr*^−/−^ recipients copied the hematological phenotype of germinal *Osmr*^−/−^ mice [96]. By the same token, this suggests that the inflammatory skin reaction observed in response to subcutaneous injection of recombinant OSM [101, 102] is unlikely to be initiated by inflammatory leukocytes in the skin but rather by direct action on keratinocytes and epidermal stem cells which abundantly express OSMR [103, 104].

RNA sequencing of HSC isolated from the BM of *Osmr*^−/−^ and wild-type mice has revealed that, in the absence of OSMR signaling, HSC have reduced expression of genes necessary for erythroid differentiation (e.g., *Gata1*, *Klf1*, *Alad*), of genes involved in progression through cell cycle such as *Ccnb2* and *Sertad1*, and of genes regulating energy supply from lipid catabolism [23••]. Again, these effects must be indirectly mediated via non-hematopoietic cells in the HSC niches since neither HSC nor their progeny express OSMR. How these BM stromal and endothelial cells subsequently regulate HSC via OSMR/OSM signaling remains unknown as RNA sequencing of non-hematopoietic BM niche cells from *Osmr*^−/−^ and wild-type mice has not been done. The endothelial cell-specific cell adhesion molecule E-selectin may be involved since OSM stimulates E-selectin expression by endothelial cells in vitro [101], *Osmr*^−/−^ mice have reduced E-selectin expression in the BM [23••], and E-selectin acts directly on fucosylated and sialylated non-canonical receptors (CD44 and CD161/PSGL1 decorated with O-glycosylations containing sialyl Lewis^a/x^ polysaccharides are the canonical receptors of E-selectin) to promote HSC proliferation and differentiation in vivo [105].

## OSM-OSMR Signaling Acts as a Brake to HSC Trafficking and Mobilization

The BM is not a tightly held compartment with a small proportion of HSPC continuously escaping into the circulation and returning to the BM. Following 3- to 6-day course of daily injections of a hematopoietic growth factor such as G-CSF or KIT ligand, large numbers of HSPC actively egress from the BM into the circulation, a process called HSPC mobilization [106]. Mobilization is used clinically to harvest large number of HSPC to transplant into patients needing autologous or allogeneic hematopoietic reconstitution and has supplanted BM aspiration as the source of transplantable HSPC since the late 1990s. G-CSF actions to mobilize HSPC are mostly indirect, and mediated by release of factors (such as neutrophil/macrophage proteases, catecholamines, and chemokines) from neutrophils, dendritic cells, macrophages, and sympathetic neurons that proteolyze or reduce expression of cell adhesion molecules and chemokines, such as VCAM-1, KIT receptor, and CXCL12, which are produced by BM stromal and endothelial cells to anchor HSPC in their niches [107, 108]. Additional mechanisms involve the production of a hypoxic environment which triggers hypoxia signaling in HSC [109].

It has been recently found that OSM protein is abundantly released from BM neutrophils following G-CSF treatment in mice and humans [23••]. This is consistent with previous observations, including that G-CSF treatment causes neutrophilia, that the rolling of neutrophils on selectin-expressing endothelial cells induces OSM release from neutrophil granules [110], and that BM endothelial cells express E-selectin even in steady-state conditions [111]. Most importantly, OSM released from BM neutrophils during the course of G-CSF administration appears to act as an endogenous brake to HSPC mobilization. Indeed, neutrophilia and HSPC mobilization in response to G-CSF or to the clinical CXCR4 antagonist plerixafor was much higher in *Osmr*^−/−^ mice compared to wild-type. Furthermore, injection of a recombinant OSM trap comprising a fusion of the extracellular domains of OSMR and GP130 also enhanced HSPC mobilization in response to G-CSF [23••]. OSM signaling restricts HSC mobilization in multiple ways by increasing E-selectin expression in HSC vascular niches, increasing HSC chemotactic response to CXCL12, and increasing expression of proteins involved in HSC cytoskeleton organization [23••]. Again these mechanisms are indirect via BM stromal cells because HSPC do not express OSMR [23••]. These in vivo results in mice are consistent with a previous finding that OSM released by neutrophils signals to endothelial cells via GP130-containing receptor complexes to increase vascular selectin expression and thereby increase selectin-mediated neutrophil rolling onto the activated endothelium [112]. These results indicate that neutrophils, while driving HSPC mobilization, also provide their own in-built endogenous brake by secreting OSM. They also suggest that pharmacological neutralization of OSM may represent a new strategy to enhance HSC mobilization, particularly in donors who mobilize poorly [23••].

## OSM and Diabetic HSC Mobilopathy

Individuals with type 1 (T1D) or type 2 (T2D) diabetes are well known to mobilize HSC poorly in response to G-CSF and this is called diabetic HSC mobilopathy [113]. Mechanistically, hyperglycemia alters BM stroma and vascular architecture and function, with increased myelopoiesis in diabetic humans and mice. In BMSC of diabetic mice, expression of the HSC retention chemokine CXCL12 is increased, and down-regulation of CXCL12, which normally occurs in response to G-CSF to enable HSC mobilization, is abated [114]. OSM has been reported to play an important role in diabetic mobilopathy in humans and mice [115]. T1D patients responded to mobilization with a greater increase in inflammatory type monocytes than healthy controls, and this was negatively correlated with CD34^+^ HPC mobilization. Likewise, streptozotocin-induced T1D mice had higher proportion of inflammatory macrophages in the BM and depletion of these macrophages restored high levels of HSC mobilization in response to G-CSF [115]. This suggested that, as in healthy mice [11], BM macrophages in diabetic mice also produce a HSC retention signal. It was found that this HSC retention signal produced by BM macrophages in T1D mice is OSM. T1D mice had abnormally high OSM concentration in BM extracellular fluids [116••]. Furthermore, injection of neutralizing anti-OSM polyclonal antibodies [115] or germinal deletion of the *Osm* gene [116••] restored CXCL12 down-regulation and HSPC mobilization in response to G-CSF. This effect of T1D and OSM on HSC mobilopathy was found to be dependent on the adaptor protein SHC1, since targeted *Shc1* deletion in hematopoietic cells in T1D mice partially corrected the HSC mobilopathy, reduced myelopoiesis, and restored the BM vascular architecture [116••]. However, blocking OSM as a therapeutic maneuver to correct diabetic HSC mobilopathy should be considered with caution because recent reports indicate that *Osm*^−/−^ mice fed a high fat diet have increased glucose intolerance [117•]. From a therapeutic viewpoint, it was also found that treatment with the clinical peroxisome proliferator activated receptor-γ (PPARγ) agonist pioglitazone down-regulated expression of OSM and CXCL12 in the BM of T1D mice and restored HSPC mobilization in response to G-CSF [118]. Likewise in humans with T2D, pioglitazone treatment increased HSPC mobilization in response to G-CSF [118].

Therefore, in both the healthy and diabetic settings, OSM acts as a brake to therapeutic HSPC mobilization in response to G-CSF by indirect actions mediated through BM stromal cells (mesenchymal and endothelial) that express OSMR. However, these mechanisms differ between healthy and diabetic animals. In healthy mice, OSM is mainly released by BM neutrophils and seems to act by up-regulating E-selectin with little effect on CXCL12 expression [23••]. In contrast, in models of T1D, OSM seems to be released mostly by BM inflammatory macrophages and up-regulate CXCL12 expression [115, 116••, 119]. The proximity of intravasating BM neutrophils to endothelial cells in response to G-CSF [120], and BM macrophages to BMSC [121] may explain the two different ways by which OSM increases HSPC retention in healthy versus diabetic BM.

Whether the actions of OSM on the osteoblast lineage might contribute to diabetic mobilopathy or to diabetic bone disease remains unknown and, to our knowledge, has not been investigated.

## Conclusion

OSM plays multiple roles in regulating the skeletal and hematopoietic tissues in part because OSM has 2 distinct GP130-linked receptors, OSMR and LIFR, which elicit biases towards different downstream signaling cascades in osteoblasts, mesenchymal progenitors, BM stromal, and endothelial cells. The production of OSM by myeloid and mesenchymal cells which are both regulators and cell components of skeletal and hematopoietic tissues illustrates the importance of OSM biology. Nuances in OSM and OSMR biology remain poorly understood because most experiments on the role of OSM and OSMR have been performed on mice with germinal deletion of either of these two genes. Now that mice with a floxed *Osm* gene are becoming available, cell-specific conditional deletion of these two genes will enable identification of the key cellular sources, including whether OSM produced by osteoblasts, macrophages, or neutrophils have different roles in regulating bone and bone marrow tissues. Likewise for *Osmr*, conditional knock-out approaches would enable a better understanding of the mesenchymal, osteoblast, and endothelial contributions of OSMR-mediated signaling. Finally, in addition to regulating blood and bone, OSM and OSMR may play important roles various inflammatory responses such as in sepsis [122, 123], muscle maintenance [92•], and epidermal stem cells in hair follicles and metabolic diseases [104, 117•] as well as in the progression and response to treatment of several malignancies [124, 125, 126•, 127].
